# Target Nuclear and Off-Target Plastid Hybrid Enrichment Data Inform a Range of Evolutionary Depths in the Orchid Genus *Epidendrum*

**DOI:** 10.3389/fpls.2019.01761

**Published:** 2020-01-29

**Authors:** Carolina Granados Mendoza, Matthias Jost, Eric Hágsater, Susana Magallón, Cássio van den Berg, Emily Moriarty Lemmon, Alan R. Lemmon, Gerardo A. Salazar, Stefan Wanke

**Affiliations:** ^1^ Departamento de Botánica, Instituto de Biología, Universidad Nacional Autónoma de México, Mexico City, Mexico; ^2^ Institut für Botanik, Technische Universität Dresden, Dresden, Germany; ^3^ Herbario AMO, Instituto Chinoin, A.C., Mexico City, Mexico; ^4^ Departamento de Ciências Biológicas, Universidade Estadual de Feira de Santana, Feira de Santana, Brazil; ^5^ Department of Biological Science, Florida State University, Tallahassee, FL, United States; ^6^ Department of Scientific Computing, Florida State University, Tallahassee, FL, United States

**Keywords:** Orchidaceae, anchored hybrid enrichment, universal probe set, off-target data, coalescent methods, phylogenomics

## Abstract

Universal angiosperm enrichment probe sets designed to enrich hundreds of putatively orthologous nuclear single-copy loci are increasingly being applied to infer phylogenetic relationships of different lineages of angiosperms at a range of evolutionary depths. Studies applying such probe sets have focused on testing the universality and performance of the target nuclear loci, but they have not taken advantage of off-target data from other genome compartments generated alongside the nuclear loci. Here we do so to infer phylogenetic relationships in the orchid genus *Epidendrum* and closely related genera of subtribe Laeliinae. Our aims are to: 1) test the technical viability of applying the plant anchored hybrid enrichment (AHE) method (Angiosperm v.1 probe kit) to our focal group, 2) mine plastid protein coding genes from off-target reads; and 3) evaluate the performance of the target nuclear and off-target plastid loci in resolving and supporting phylogenetic relationships along a range of taxonomical depths. Phylogenetic relationships were inferred from the nuclear data set through coalescent summary and site-based methods, whereas plastid loci were analyzed in a concatenated partitioned matrix under maximum likelihood. The usefulness of target and flanking non-target nuclear regions and plastid loci was assessed through the estimation of their phylogenetic informativeness. Our study successfully applied the plant AHE probe kit to *Epidendrum*, supporting the universality of this kit in angiosperms. Moreover, it demonstrated the feasibility of mining plastome loci from off-target reads generated with the Angiosperm v.1 probe kit to obtain additional, uniparentally inherited sequence data at no extra sequencing cost. Our analyses detected some strongly supported incongruences between nuclear and plastid data sets at shallow divergences, an indication of potential lineage sorting, hybridization, or introgression events in the group. Lastly, we found that the per site phylogenetic informativeness of the *ycf1* plastid gene surpasses that of all other plastid genes and several nuclear loci, making it an excellent candidate for assessing phylogenetic relationships at medium to low taxonomic levels in orchids.

## Introduction

Powerful hybrid enrichment strategies (HES), a toolset for selectively capturing genomic regions of interest prior to sequencing (Summerer, 2009; Mamanova et al., 2010; Lemmon and Lemmon, 2013), are increasingly being applied to plant phylogenomics, boosting generation of massive sequence data and, therefore, opening exciting new possibilities for plant evolutionary studies. Previously HES applied to angiosperm phylogenomics include an assortment of nuclear-exon and organelle (plastid) enrichment methods targeting a range of taxonomic levels and lineages. Some differences among these techniques include 1) how capture probes are designed, e.g., whether they target dozens (de Sousa et al., 2014) to thousands (Mandel et al., 2014; Weitemier et al., 2014) of more or less conserved genomic regions; 2) if the focus is taxon-specific (e.g., *Gossypium*, Grover et al., 2015; *Sabal*, Heyduk et al., 2016; *Helianthus*, Stephens et al., 2015) or has a wider taxonomic scope (e.g., angiosperms, Johnson et al., 2019; Zingiberales, Sass et al., 2016; eudicots, Stull et al., 2013); 3) the targeted genome i.e., nuclear (de Sousa et al., 2014; Mandel et al., 2014; Grover et al., 2015) or plastid (Stull et al., 2013). The mitochondrial genome has only begun to be targeted for angiosperm phylogenomic studies (Li et al., 2019). Due to the relatively small size of the plastid genome and its relatively high copy number per cell, assembling complete plastomes (Stull et al., 2013; Sass et al., 2016) or large portions of them (Heyduk et al., 2016) can easily be achieved. Additionally, off-target plastid reads can potentially be recovered after nuclear enrichment, providing a valuable added source of orthologous, uniparentally inherited sequence data (Weitemier et al., 2014; Stephens et al., 2015; Nikolov et al., 2019) to complement nuclear data.

Plant anchored hybrid enrichment (AHE; Buddenhagen et al., 2016) is a highly efficient HES that has rapidly been applied to several angiosperm lineages and an ample range of taxonomic levels (Cardillo et al., 2017; Fragoso-Martínez et al., 2017; Mitchell et al., 2017; Shahi Shavvon et al., 2017; Wanke et al., 2017; Léveillé-Bourret et al., 2018; Crowl et al., 2019; Kriebel et al., 2019). The design of plant AHE probes was based on complete and low-coverage genomes from a variety of species representing all main angiosperm lineages, being potentially applicable to any flowering plant lineage. Since less conserved flanking regions can be captured along with the conserved target regions, retrieved loci can also inform at a range of evolutionary depths.

In this work, we explore the utility of the plant AHE method to resolve phylogenetic relationships in the orchid genus *Epidendrum* (subtribe Laeliinae, tribe Epidendreae, subfamily Epidendroideae), which currently includes over 1,500 Neotropical species exhibiting a great degree of vegetative and reproductive variation, habitat preferences, and ecological interactions (Pérez García, 1993; van den Berg, 2000; Hágsater and Soto-Arenas, 2005; Pinheiro and Cozzolino, 2013). Previous molecular phylogenetic studies of this genus have been based on a single [i.e., nuclear ribosomal internal transcribed spacer (ITS) region; van den Berg et al., 2000] or a few markers (ITS plus plastid *atpI-atpH*, *rpl32-trnL*, *rps16*, *trnH-psbA*, *trnL-trnF*, *trnS-trnfM*, and *matK-trnK* regions: Hágsater and Soto-Arenas, 2005; van den Berg et al., 2009; Quiroga-González, 2017; Pagnussat Klein et al., 2019) obtained *via* Sanger sequencing. Those studies indicated that *Epidendrum* (currently including: *Oerstedella*, *Amblostoma*, *Lanium*, and *Nanodes*; Hágsater and Soto-Arenas, 2005) forms a clade, known as the *Epidendrum* Alliance, together with the genera *Orleanesia*, *Barkeria*, *Caularthron* (van den Berg et al., 2000; van den Berg et al., 2009), and *Microepidendrum* (Hágsater and Soto-Arenas, 2005).

The study of Hágsater and Soto-Arenas (2005) provided a general phylogenetic framework recognizing some major clades of *Epidendrum*. However, support for these major clades and, in general, for backbone relationships remains low due to the lack of informative data. Several fundamental questions persist, such as whether or not the genus is monophyletic, and the identity of its sister lineage (Hágsater and Soto-Arenas, 2005). A well-resolved and strongly supported phylogenetic framework will also facilitate the establishment of a formal infrageneric classification.

For these reasons, *Epidendrum*, along with a careful selection of outgroup taxa, conforms an ideal system to test the technical implementation of the AHE and its performance in resolving and supporting phylogenetic relationships at intermediate to shallow taxonomic levels. Because no orchid representatives were originally included in the set of reference species used to design the plant AHE capture probes, *Epidendrum* represents an excellent group for testing the general applicability of this technique in angiosperms. Additionally, we here explore the feasibility of mining off-target plastid genes from targeted nuclear enrichment data to increase the amount of potentially informative data, as well as to generate an uniparentally inherited dataset without additional sequencing effort. Our aims are to 1) test the technical viability of applying the plant AHE probe set (Angiosperm v.1 probe kit) to *Epidendrum* and outgroup species of subtribe Laeliinae, 2) mine plastid protein coding genes from off-target reads; and 3) evaluate the performance of the target nuclear AHE and off-target plastid loci in resolving and supporting phylogenetic relationships at a range of taxonomical depths within both Laeliinae and *Epidendrum*. In order to account for potential nuclear gene tree discordance, coalescent summary (Zhang et al., 2018) and site-based (Chifman and Kubatko, 2014) methods were performed for phylogenetic inference. Plastid data was analyzed under a concatenated partitioned approach under maximum likelihood. The phylogenetic utility of loci was assessed through the phylogenetic informativeness method of Townsend (2007).

## Materials and Methods

### Taxon Sampling and Deoxyribonucleic Acid Extraction

Our taxon sampling comprised 18 *Epidendrum* species representing the two main clades previously recognized by Hágsater and Soto-Arenas (2005), including five species previously shown to be closely related to each other as members of the “*Epidendrum anisatum* group” (Quiroga-González, 2017), as well as one species each of the genera *Arpophyllum*, *Barkeria*, *Broughtonia*, and *Caularthron* of subtribe Laeliinae. Such sampling strategy permitted us to assess the phylogenetic utility of our nuclear and plastid data both among major clades, as well as closely-related species of *Epidendrum*. Phylogenetic trees were rooted with *Pleurothallis cardiothallis* of subtribe Pleurothallidinae, because this subtribe was recovered in previous phylogenetic analyzes of Orchidaceae as sister to subtribe Laeliinae (**Table 1**; Chase et al., 2015). Genomic DNA of one individual per species was extracted from fresh or silica-gel dried leaf tissue with the cetyl trimethylammonium bromide (CTAB) method of Doyle and Doyle (1987) modified to include RNase A (Qiagen, 100 mg/ml) and proteinase K (Thermo Scientific, 1 mg/ml) during incubation phases. A NanoDrop 2000/2000c spectrophotometer (Thermo Scientific) was used to ensure a minimum amount of 2.3 µg of DNA per sample with 260/280 and 230/260 purity ratios ≥0.84. Agarose (2.0%) test gels were run for 90 min at 120 V to confirm the presence of bands of high molecular weight and visual assessment of DNA fragmentation.

**Table 1:** Taxon sampling and voucher information including collector, and collection number (herbarium code as in http://sweetgum.nybg.org/science/ih/).

Function	Subtribe	Taxon	Voucher information	Lab #
Outgroup	Pleurothallidinae	*Pleurothallis cardiothallis*	Soto Arenas, 3679 (AMO)	E100
	Laeliinae	*Arpophyllum giganteum*	Soto Arenas, 6858 (AMO)	E106
		*Barkeria melanocaulon*	Jiménez Machorro, 2445 (AMO)	E094
		*Broughtonia sanguinea*	Brieger, 14440 (ESA)	E218
		*Caularthron bicornutum*	van den Berg, 2997 HUEFS)	E217
Ingroup		*Epidendrum anisatum*	Hágsater, 14559 (AMO)	E026
		*E. ciliare*	Hágsater, 9468 (AMO)	E058
		*E. cusii*	Salazar Chávez, 7467 (AMO)	E432
		*E. gasteriferum*	Salazar Chávez, 7566 (AMO)	E147
		*E. juergensenii*	Salazar Chávez, 7867 (AMO)	E145
		*E. lacertinum*	Soto Arenas, 9087 (AMO)	E063
		*E. longicaule*	Jiménez Machorro, 2763-B (AMO)	E013
		*E. magnoliae*	Hágsater, 14573 (AMO)	E079
		*E. mathewsii*	Hágsater, 13559 (AMO)	E070
		*E. matudae*	Salazar Chávez, 7468 (AMO)	E008
		*E. nocturnum*	Hágsater, 13963 (AMO)	E065
		*E. octomerioides*	Hágsater, 13804 (AMO)	E017
		*E. parkinsonianum*	Hágsater, 14582 (AMO)	E042
		*E. propinquum*	Hágsater, 14591 (AMO)	E015
		*E. sophronitoides*	Hágsater, 14552 (AMO)	E048
		*E. succulentum*	Salazar Chávez, 6723 (AMO)	E435
		*E. summerhayesii*	Hágsater, 14587 (AMO)	E044
		*E. trialatum*	Hágsater, 14558 (AMO)	E022

### Plant Anchored Hybrid Enrichment

The Angiosperm v.1 probe kit (Buddenhagen et al., 2016) was used for enrichment. Details of how this probe kit was designed have been extensively explained elsewhere (Buddenhagen et al., 2016; Fragoso-Martínez et al., 2017; Wanke et al., 2017). A recent study in the orchid genus *Lepanthes* (subtribe Pleurothallidinae; Bogarín et al., 2018) applied a modified version of this kit targeting longer and potentially more variable loci so that the retrieved markers were more suitable for population level studies. Since the aim of the present study is to investigate phylogenetic relationships at higher taxonomic ranks (i.e., above the species level) among species of *Epidendrum* and to other genera of Laeliinae and Pleurothallidinae, we applied the original version of the plant AHE probe set of Buddenhagen et al. (2016).

Library preparation and enrichment was performed in the Center for Anchored Phylogenomics at Florida State University (www.anchoredphylogeny.com) as described in Fragoso-Martínez et al. (2017). Up to 16 samples were pooled per enrichment reaction. Enriched libraries were sequenced on one PE150 Illumina HiSeq2000 lane at the Translational Science Laboratory in the College of Medicine at Florida State University.

### Read Processing, Assembly, Orthology Assessment, and Alignment of Nuclear Loci

The CASAVA v. 1.8 pipeline was used to filter low-quality raw reads applying a high-chastity setting. Filtered reads were demultiplexed and those failing to match exactly any of 13 in-house developed indexes were discarded. The code and parameter settings used for read merging, assembly, orthology assessment, and alignment of nuclear loci is available as **Supplementary Material S1**.

A conservative method, designed to prevent read merging at highly repetitive regions, was performed following Rokyta et al. (2012). Merged and unmerged reads were assembled with the quasi-*de novo* assembler described by Prum et al. (2015). In the first step of this assembler, reads are mapped to conserved regions of the target loci with three distant species (*Arabidopsis thaliana*, *Billbergia nutans*, and *Carex lurida*) being selected as references from the set of species used by Buddenhagen et al. (2016) in the probe set design. The assembler uses a library of spaced kmers (k = 20), derived from the conserved sites of the alignments of the three reference species, to determine which target locus a particular read is derived from. Preliminary candidate locus matches are identified if a minimum of 17 (out of 20) matches are found between a spaced kmer and the read. Then the read is compared to the reference sequence from which the kmer was derived and if 55 bases out of 100 bases surrounding the kmer match between the read and the kmer, the read is said to be a confirmed match. Approximate alignment position of reads mapped this way was estimated using the position of the spaced 20-mer. In the second step reads assembled in the first step are used to create a hash table of 60-mers that serve as references to extend the assembly into more variable flanking regions. The two assembly steps are used to traverse repeatedly the read files until no additional reads are mapped.

One consensus sequence with heterozygous sites coded as IUPAC ambiguity codes was produced for each species per orthologous locus. Unambiguous bases were called if no polymorphism was observed or if polymorphisms could be attributed to sequencing errors. Bases were called as N if coverage was below 10. In order to prevent cross contamination and potential sequencing errors in index reads, assembly contigs with less than 30 reads were discarded.

For orthology assessment, consensus sequences were grouped by locus and a distance matrix was generated with the pairwise distance between two sequences as the percent of 20-mers found in both sequences. Based on these distance matrices, sequences were clustered using the neighbor-joining algorithm (Saitou and Nei, 1987). If gene duplication, low coverage and contamination are absent, each locus should produce a single cluster with a single sequence per species. If more than one cluster was retrieved per locus, each cluster of orthologs was considered as a different locus and separated from the other(s). Clusters with less than 50% of the target species were discarded in order to reduce the effect of missing data. Pre-alignments were produced using MAFFT v.7.023b (Katoh and Standley, 2013) and subsequently trimmed following Prum et al. (2015) and Hamilton et al. (2016) criteria to generate the final nuclear alignments. All methods described in this section were performed by the Center for Anchored Phylogenomics.

### Read Processing, Assembly, and Alignment of Plastid Loci

The raw data was assembled using CLC Genomics Workbench v.11.0 (https://www.qiagenbioinformatics.com/). A *de novo* assembly for each of the accessions was created, allowing for automatic word and bubble size, as well as an auto-detection of paired distances. To solve potential mis-assemblies or inconsistencies in the assemblies, readmapping files were extracted after mapping the reads back to the contigs. Plastid protein coding genes and ribosomal RNAs (rRNAs) were identified using BLAST and automatically aligned to a reference using the Python Workflow for 1kp Assemblies (written by Wesley K. Gerelle, University of British Columbia; https://github.com/wesleykg/1kp_workflow), combining a BLAST search (e-value = 1e−20) and an alignment of the hits back to the reference (MUSCLE, standard settings). The reference file was prepared by extracting sequences of 79 protein coding and four rRNA genes (a total of 83 loci) from the plastome of *Masdevallia coccinea* (NC_026541.1). The BLAST results in combination with the readmapping files were then used to extract the genes from the target species. Translocation between the plastid and mitochondrial genomes has been reported for genes of the *ndh* family in some Epidendroideae representatives (Lin et al., 2015); therefore, the complete *ndh* gene family (11 genes) was excluded from further analyses to prevent a possible mixture of mitochondrial and plastid copies for these genes. After exclusion of the *ndh* genes, 72 plastid loci were the targeted in our mining strategy. Sequences and BLAST results were visualized using AliView v.1.18.1 (http://ormbunkar.se/aliview/; Larsson, 2014), and readmapping files using Tablet v.1.17.08.17 (https://ics.hutton.ac.uk/tablet/). Single gene alignments were manually created using AliView v.1.18.1 and alignments were concatenated in Geneious v.11.1.5 (https://www.geneious.com).

### Tree Reconstruction

Coalescent summary and site-based methods were selected for phylogenetic inference of the nuclear data set to accommodate potential gene-tree heterogeneity, for instance, caused by incomplete lineage sorting (Maddison, 1997; Degnan and Rosenberg, 2009), and because these methods are known to handle appropriately large numbers of loci and/or taxa (Chifman and Kubatko, 2014; Molloy and Warnow, 2017). Summary methods use gene trees as input for species tree estimation, so that gene trees are summarized into a species tree (Liu et al., 2009; Zhang et al., 2018). However, summary methods are sensitive to gene-tree estimation errors, for instance, those resulting from missing data or low number of informative sites per individual locus (Roch and Warnow, 2015). Due to these known caveats, and to confirm summary methods results, we also applied site-based methods which directly infer the species tree from site patterns in the alignments of the different loci (Chifman and Kubatko, 2014), thus circumventing the difficulties associated to gene tree estimation. Coalescence summary analyses were performed in ASTRAL-III (Zhang et al., 2018), estimating branch support through local posterior probabilities (Sayyari and Mirarab, 2016). Input nuclear gene trees for ASTRAL-III were generated using a workflow created in Geneious that runs in series maximum likelihood (ML) analyses on all loci within a folder with the implemented RAxML (Stamatakis, 2006) plugin, applying a GTR + Γ model as recommended in the RAxML manual. Twenty searches for the best ML tree were performed per locus and clade support was assessed with 1,000 bootstrap replicates. Node support was indicated on the best ML trees using a modification of the “applyRAxML2AllFilesInDirectory.pl” Perl script (https://github.com/stamatak/standard-RAxML/tree/master/usefulScripts). Gene trees were further manipulated to collapse nodes with bootstrap support (BS) < 33 (**Supplementary Material S2**), since this has been shown to increase accuracy of the species tree estimation in ASTRAL (Sayyari and Mirarab, 2016). Nodes were collapsed with the program TreeCollapseCL 4 (Emma Hodcroft, http://emmahodcroft.com/TreeCollapseCL.html).

Coalescent site-based analyses were performed in SVDquartets (Chifman and Kubatko, 2014) as implemented in PAUP 4.0a 165 (Swofford, 2003). The input was a NEXUS file containing the concatenated nuclear matrix partitioned by locus. Up to 100,000 randomly sampled quartets were evaluated and 1,000 bootstrap replicates were performed.

Phylogenetic inference of plastid data was performed under a concatenated approach with maximum likelihood (ML). PartitionFinder2 v.2.1.1 (Lanfear et al., 2017) was used to determine the best-fit subset partitioning scheme of the concatenated matrix of all selected plastid loci (after excluding the *ndh* genes as explained above). One hundred independent searches for the best tree were run on the concatenated partitioned plastid matrix and node support was estimated through 1,000 bootstrap replicates on RAxML v.8.2.10 (Stamatakis, 2014).

### Estimation of Phylogenetic Informativeness

Performance of plastid and nuclear loci, the latter divided into target and flaking non-target regions, in resolving a range of evolutionary depths within *Epidendrum* and subtribe Laeliinae was estimated with the phylogenetic informativeness method of Townsend (2007). Position of nuclear target and flaking non-target regions was determined on final alignments, after trimming ends and ambiguously aligned regions. Flanking regions falling outside the target region were considered non-target regions. Branch lengths of the topology obtained from the nuclear SVDQuartets analysis were optimized in RAxML, applying a GTR + Γ model and the combined data matrix partitioned by locus. The resulting phylogenetic tree was then made ultrametric, assigning time 0 for tree tips and time 1 to the root, in R v.3.5.0 with the function “chronopl” of the APE package (Paradis et al., 2004), setting lambda to 0.0. This modified ultrametric tree, along with a combined partitioned matrix, was uploaded to the PhyDesign web application (López-Giráldez and Townsend, 2011). Input partitions for this combined matrix corresponded to each of the plastid loci, nuclear target regions and nuclear non-target flaking regions. Substitution rates were estimated with the HyPhy program (Pond et al., 2005) applying a generalized time reversible (GTR) evolutionary model by inputting base frequencies and substitution rate matrix obtained from the analysis of the combined data set with Phyml (Guindon and Gascuel, 2003) in JModelTest2 v. 2.1.6 (Darriba et al., 2012). Net phylogenetic informativeness profiles were plotted for each individual plastid and nuclear locus and contrasted against the reference ultrametric tree. Additionally, maximum net phylogenetic informativeness (PImax) was recorded for each locus.

## Results

### Nuclear Gene Capture

**Figure 1:**
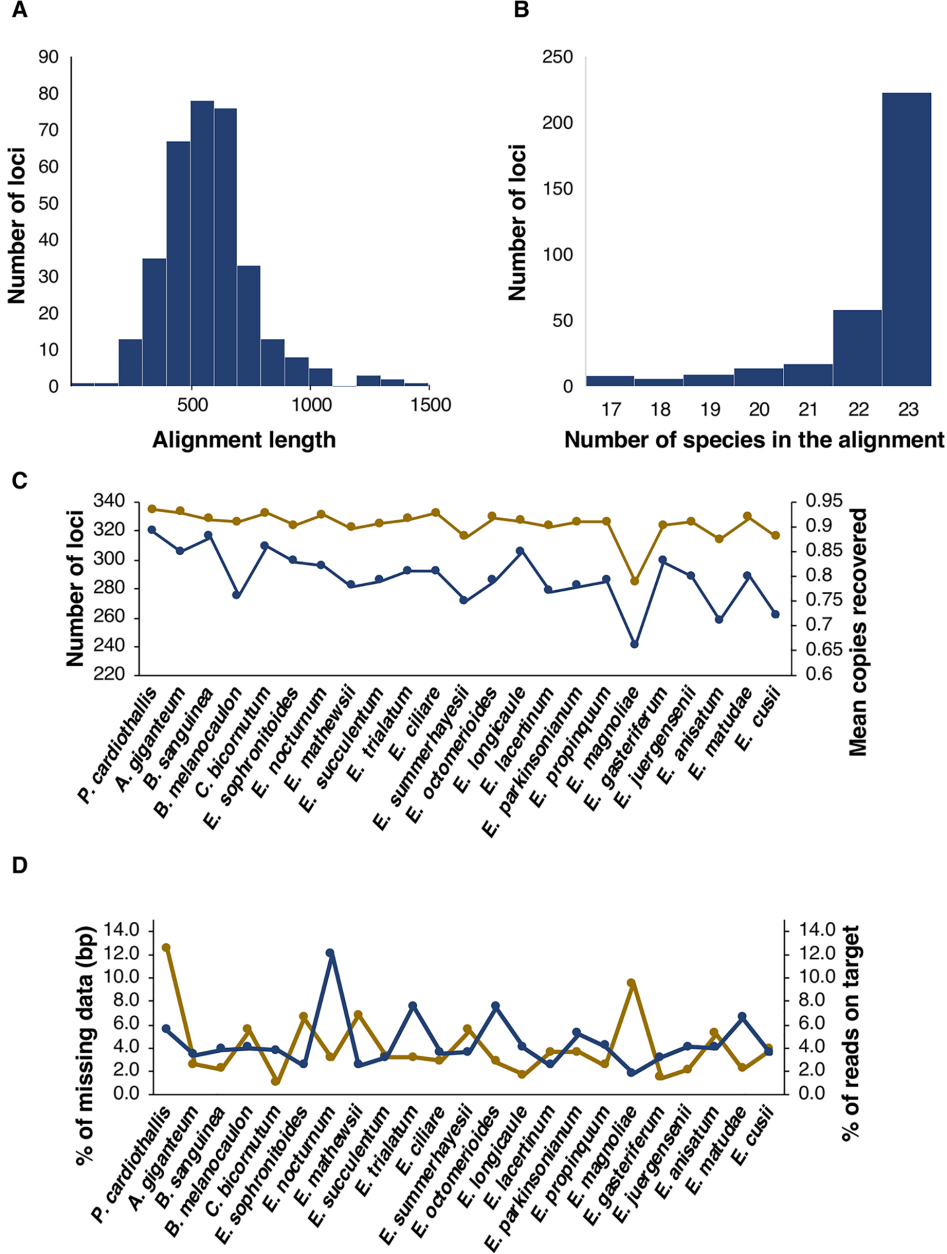
Attributes of retrieved nuclear loci. **(A, B)** Histograms showing length and number of species in the alignments, respectively. **(C)** Number of loci (yellow points and lines) and mean copies recovered (blue points and lines) per species. **(D)** % of missing data per species, including bases called as N plus missing flanking regions of loci, in terms of number of base pairs (bp, yellow points and lines) and % of reads on target per species (blue points and lines).

From the 517 loci included in the plant AHE kit, 316 were recovered with a single copy after the entire nuclear pipeline for our taxonomic sample. Additionally, eight loci had two copies and one had three copies, therefore the total nuclear data set was composed of 335 orthologs (**Supplementary Material S3**). Alignment length varied from 163 to 1,495 bp, with a mean of 581 bp (**Figure 1A**). Complete taxon sampling was achieved for 223 (67%) of the nuclear loci, 58 (17%) had one missing species, 17 (5%) had two and 37 (11%) had three or more missing species (**Figure 1B**). Within Laeliinae, the number of recovered loci per species ranged from 333 in *Arpophyllum giganteum* to 285 in *Epidendrum magnoliae*. As a result of our clustering step for orthology assessment, species can recover no copies, a single copy, or more than one copy. When considering the average number of recovered copies per species across all nuclear loci, representatives of Laeliinae ranged from 0.88 in *Broughtonia sanguinea* to 0.66 in *E. magnoliae* (**Figure 1C**).

The concatenated nuclear dataset had 194,841 sites of which 13.86% were variable (**Table 2**). In this matrix *P. cardiothallis* and *E. magnoliae* showed the highest proportion of missing data (i.e., proportion of bases called as N plus missing flanking regions; 12.5 and 9.5%, respectively), whereas all other species ranged from 6.8% in *Epidendrum mathewsii* to 1% in *Caularthron bicornutum*. Percentage of reads on target ranged from 1.8 in *E. magnoliae* to 12 in *Epidendrum nocturnum* (**Figure 1D**).

**Table 2:** Analyzed dataset characteristics.

	Nuclear	Plastid	Combined
**# of retrieved loci**	335	72	407
**Alignments length range (bp)**	163-1495	90-6990	163-6990
**# (%) alignments with 23 spp.**	223 (66.56)	66 (91.66)	289 (71)
**# total sites**	194,841	63,421	258,262
**# (%) variable sites**	27,006 (13.86)	2610 (4.11)	29,616 (11.46)

### Mined Plastid Regions

**Figure 2:**
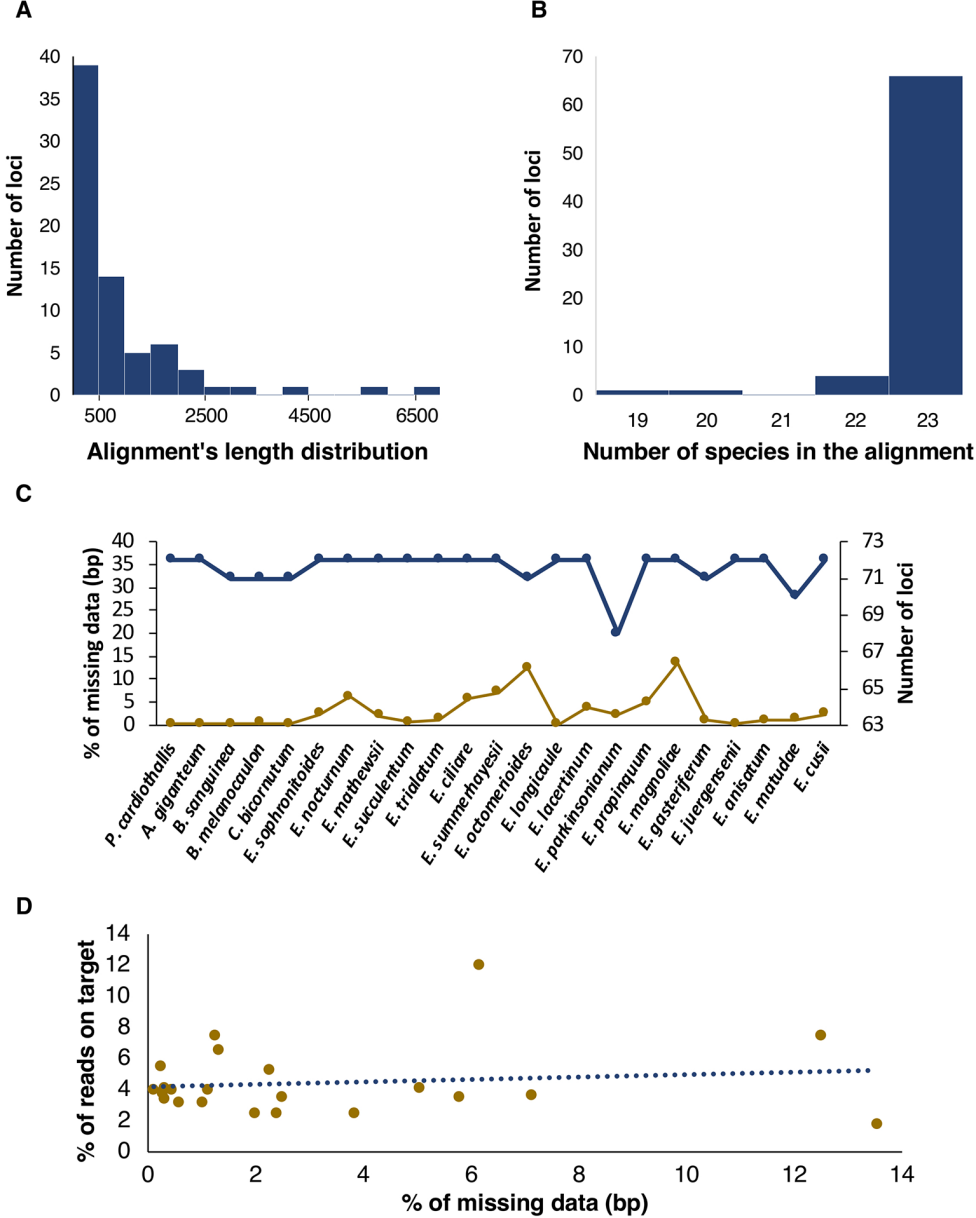
Attributes of retrieved plastid loci. **(A**, **B)** Histograms showing length and number of species in the alignments, respectively. **(C)** Missing data, including bases called as N plus missing flanking regions of loci, in terms of base pairs (bp, yellow points and lines) and number of loci (blue points and lines) per species. **(D)** % of reads on target as function of % of missing data, in terms of bp.

A total of 68 plastid protein coding genes and four rRNAs (72 loci in total) were mined successfully for our taxon sampling. Length of individual plastid alignments varied widely, from 90 bp in the *petN* gene to 6,990 bp in the *ycf2* gene (**Figure 2A**). We were able to mine most plastid loci for the complete sample of taxa (66 out of 72); four loci were recovered for 22 species and the remaining two in 20 and 19 species, respectively (**Figure 2B**). The number of recovered loci per species range from 72 (in 16 of the 23 spp.) to 68 in *Epidendrum parkinsonianum* (**Figure 2C**).

The aligned length of the concatenated plastid dataset (**Supplementary Material S4**) was 63,421 bp, from which 2,610 bp (4.11%) were variable (**Table 2**). *E. magnoliae* and *Epidendrum octomerioides* had the highest proportion (13.5 and 12.5%, respectively) of missing data and the remaining species ranged from 7.1% (*Epidendrum summerhayesii*) to 0.1% (*Epidendrum longicaule*) (**Figure 2C**). In general, percentage of missing data increased with the percentage of reads on target (**Figure 2D** and **Supplementary Material S5**).

### Phylogenetic Relationships Within Laeliinae and *Epidendrum*

No strongly supported incongruences were detected between the inference methods (SVDQuartets *vs*. ASTRAL) applied to the nuclear dataset (**Figure 4**). In contrast, six strongly supported incongruences were retrieved between the nuclear ASTRAL (**Supplementary Material S6**) and the plastid RAxML (**Supplementary Material S7**) trees involving the position of *Epidendrum sophronitoides*, *E. nocturnum*, *Epidendrum lacertinum*, *Epidendrum juergensenii*, *E. anisatum*, and *Epidendrum cusii*. When comparing the nuclear SVDQuartets (**Supplementary Material S8**) and the plastid RAxML tree, only three of the previously mentioned incongruences were maintained as strongly supported, including the relationships of *E. lacertinum, E. anisatum*, and *E. cusii* (**Figure 4**).

**Figure 3:**
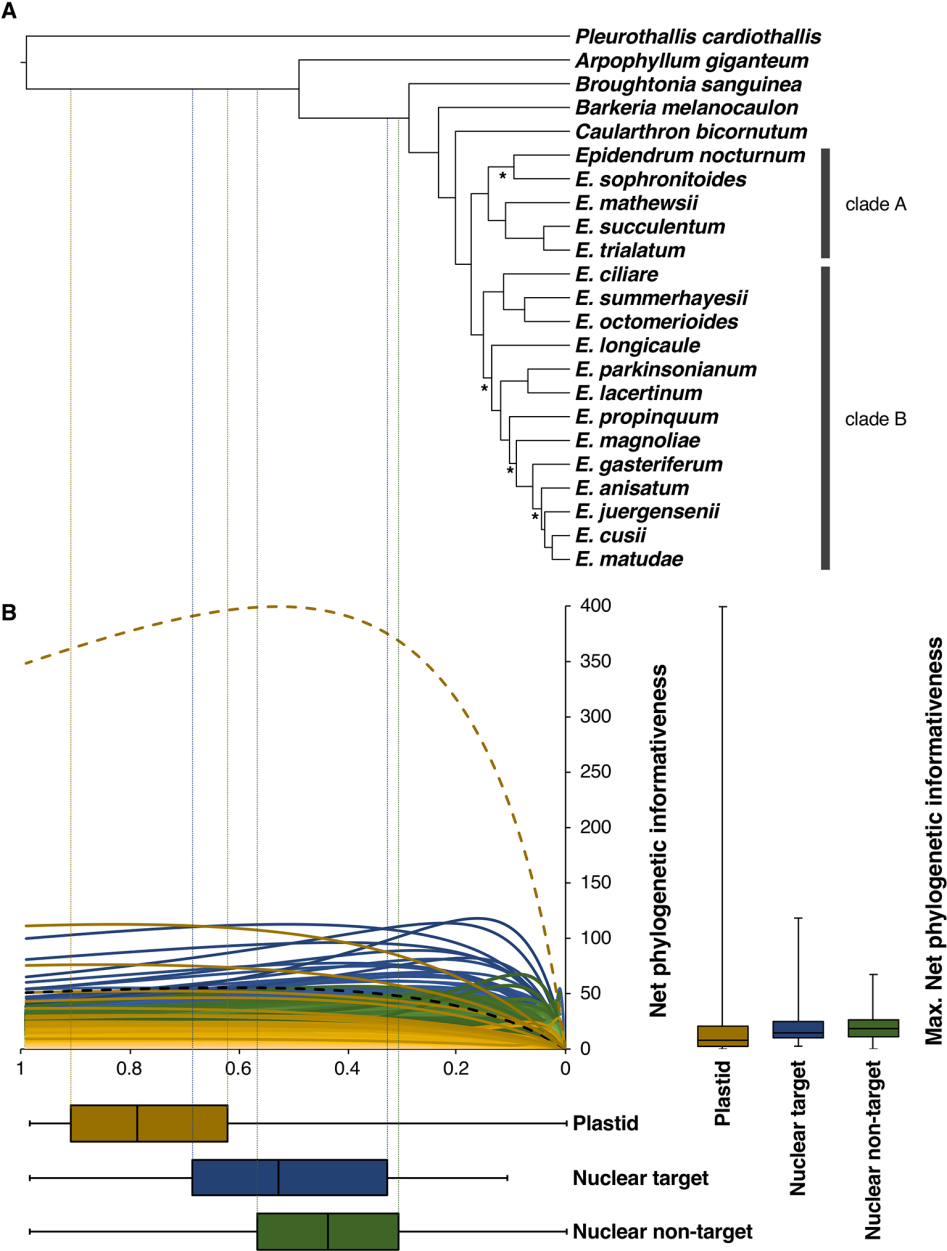
**(A)** Topology obtained in the nuclear SVDQuartets analysis with branch lengths optimized in RAxML and posteriorly converted to ultrametric (see *Materials and Methods* section). Nodes denoted by an asterisk (*) received BS < 85%. **(B)** Net phylogenetic informativeness profiles of nuclear target (light to dark blue), nuclear non-target (light to dark green), and plastid (light to dark yellow) partitions. Yellow and black dashed curves correspond to the *ycf1* and *matK* genes, respectively, discussed in the main text. Distribution of loci maximum net phylogenetic informativeness values and time at which these values were reached is shown with quantiles 2 and 3 to the right and below the informativeness profiles, respectively. Whiskers denote maximum and minimum values. Time scale of the informativeness profiles match that of the ultrametric tree in **(A)**. Vertical dotted lines denote the divergence times at which target (blue) and non-target (green) nuclear and plastid (yellow) partitions were more informative.

**Figure 4:**
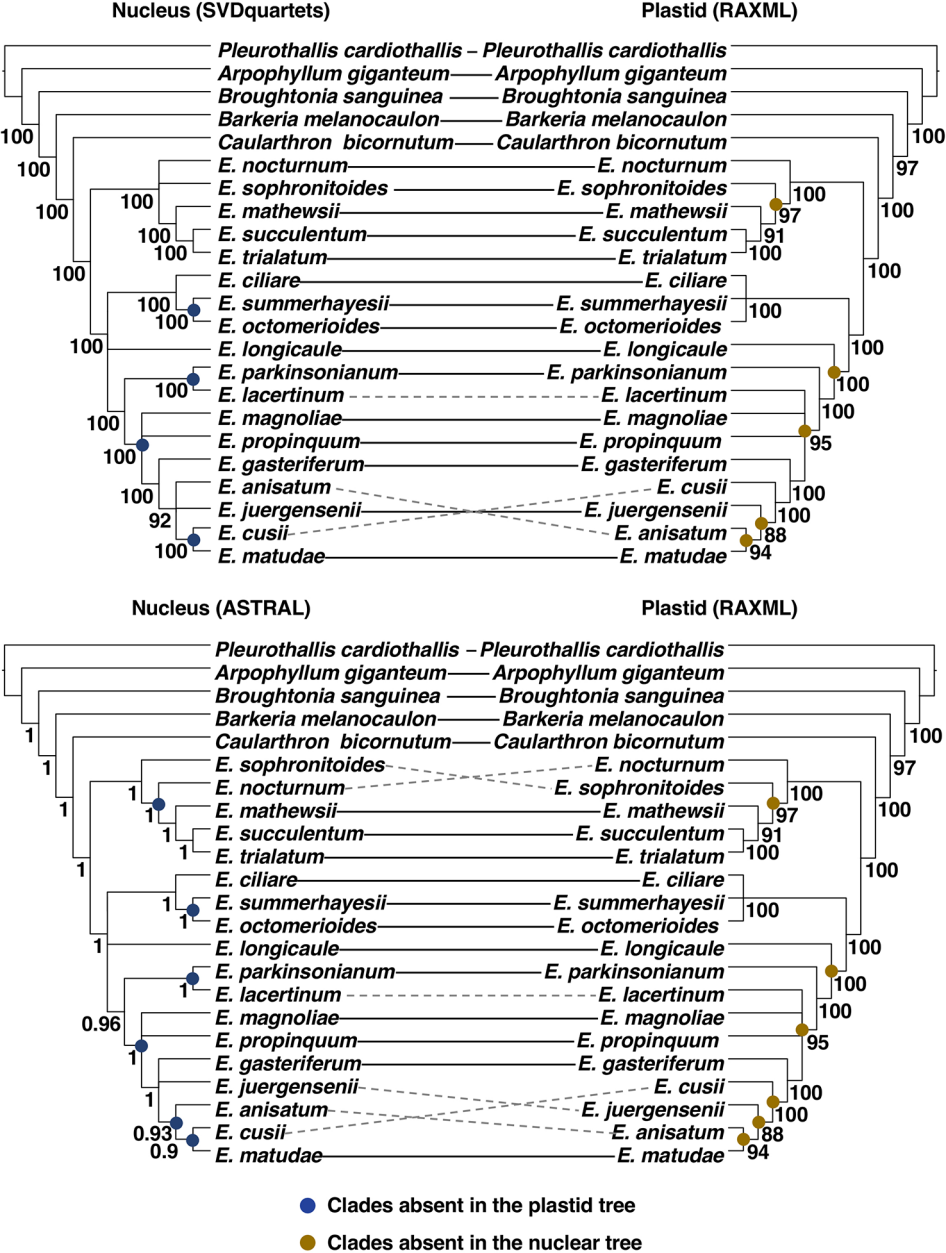
Comparison between topologies obtained from the analyses of the nuclear and plastid data sets. Continuous lines connecting names of terminal indicate congruence between topologies, whereas dotted lines indicate strongly supported (BS > 85 or LPP > 0.85) incongruencies. Blue full circles at the internal nodes of the nuclear trees indicate clades absent in the plastid tree and yellow full circles at the internal nodes of the plastid tree indicate clades absent in the nuclear trees. For ease of visualization trees were converted to cladogram and nodes with BS < 85 or LPP< 0.85 were collapsed.

Due to the higher congruence between the nuclear SVDQuartets and plastid RAxML analyses (**Figure 4**), phylogenetic relationships will be described based on the nuclear SVDQuartets tree (**Figure 3A**), where all but the already indicated relationships received BS > 85. Within subtribe Laeliinae, *A. giganteum* was recovered as sister to all other species, followed by a grade consisting of *B. sanguinea*, *Barkeria melanocaulon,* and *C. bicornutum.* The genus *Epidendrum* was found to be monophyletic and, within it, two main clades were recovered. One consists of a sister pair in which one clade includes *E. sophronitoides* sister to *E. nocturnum* (BS = 23) and the other includes *E. mathewsii*, *Epidendrum succulentum*, and *Epidendrum trialatum* as successive sisters (clade A; **Figure 3A**). The other clade consists of a sister pair in which one lineage contains *Epidendrum ciliare* as sister to the clade of *E. summerhayesii* and *E. octomerioides*, and the other lineage contains *E. longicaule* (BS = 49) as sister to a clade where [*E. lacertinum–E. parkinsonianum*] are sister to a grade of *Epidendrum propinquum*, *E. magnoliae* (BS = 52), *Epidendrum gasteriferum, E. anisatum*, *E. juergensenii* (BS = 51), and *Epidendrum matudae* sister to *E. cusii* (clade B; **Figure 3A**).

### Phylogenetic Informativeness of Nuclear and Plastid Loci

Net phylogenetic informativeness widely varied across loci (**Figure 3**). Nuclear target and flanking non-target regions showed profiles with steep increases corresponding to the shallowest divergences and gradual decreases towards the root, as well as profiles with rather flat curves. With few exceptions, plastid loci showed lower and mostly flat curves, having a gradual increase and an attenuated decrease towards the root. Maximum net phylogenetic informativeness values were in general higher for nuclear partitions, both target and non-target regions (median 14.71 and 18.56, respectively), compared to plastid loci (median 7.98). However, the *ycf1* gene strongly deviated from this general pattern showing 399.45 of maximum net phylogenetic informativeness. When the length of the markers is considered (per site phylogenetic informativeness), the *ycf1* gene is not only the plastid marker with the highest per site phylogenetic informativeness, but it also surpassed the informativeness of 40 nuclear partitions (data not shown). Most nuclear partitions reached their maximum net phylogenetic informativeness closer to the present, with median value of 0.44 for flanking non-coding regions and 0.53 for target regions, compared to plastid markers that had median value of 0.79 (**Figure 3B**).

## Discussion

### Performance of Nuclear Hybrid Enrichment

Our study is the first one to apply the Angiosperm v.1 probe kit to the orchid genus *Epidendrum*. A substantial proportion (ca. 63%) of the original targeted nuclear loci could be captured and used for phylogenetic inference. To our knowledge, eight previous studies have applied this capture kit to angiosperm lineages (**Table 3**). A general trend is observed in these studies, where larger evolutionary distance of studied taxa to the closest reference lineage used for kit design results in reduced loci recovery (including paralogs; **Supplementary Material S9**). *Epidendrum* diverged ~115 mya from the closest reference species, *Phoenix dactylifera* (Arecaceae, Arecales; Magallón et al., 2015). Two previous studies in the family Proteaceae (Cardillo et al., 2017; Mitchell et al., 2017) showed a slightly larger evolutionary distance between their focal groups and their respective reference species (~117 mya) than *Epidendrum*. However, those studies recovered a larger number of loci (498 and 450, respectively). This deviation from the general trend could be attributed to several factors, including potential loci conservation within the order Proteales since a reference species within this order was available, differences in genomic DNA (gDNA) isolates quality, and/or sequencing depth. Another possible explanation is the retrieval of a larger number of paralogs that sum up to the total number of retrieved loci. However, information about how many multicopy loci were recovered has not been consistently reported in previous studies. In *Epidendrum*, only eight loci were recovered as multicopy and the mean copies recovered per species was less than 0.88, suggesting that duplication in this set of loci is not a common process in this lineage. Regardless, the existence of a relatively large evolutionary distance (~115 mya) between *Epidendrum* and its closest monocot reference species supports the claims of Buddenhagen et al. (2016) and Wanke et al. (2017) that the Angiosperm v.1 kit is universally applicable among angiosperms.

**Table 3:** Previous studies applying the plant anchored hybrid enrichment (AHE) method (Buddenhagen et al., 2016) sorted by divergence times between the focal group and the closest set of reference species used in the kit design.

Study	Focal group (family, order)	Closest set of reference species (family, order)	Divergence time (Ma)	# retrieved loci*
Fragoso-Martínez et al. (2017)	*Salvia* subg. Calosphace (Lamiaceae, Lamiales)	*Mimulus guttatus* (Phrymaceae, Lamiales)	40.3^1^	448
Kriebel et al. (2019)	*Salvia* (Lamiaceae, Lamiales)	*Mimulus guttatus* (Phrymaceae, Lamiales)	40.3^1^	498
Shahi Shavvon et al. (2017)	*Oxytropis* (Fabaceae, Fabales)	*Medicago truncatula* and *Glycine max* (Fabaceae, Fabales)	68^2^	527
Léveillé-Bourret et al. (2018)	Cariceae–Dulichieae–Scirpeae clade (Cyperaceae, Poales)	*Carex lurida*, *C. tenax,* and *Rhynchospora chalarocephala* (Cyperaceae, Poales)	88^2^	462
Crowl et al. (2019)	*Quercus* (Fagaceae, Fagales)	*Cucumis sativus* (Cucurbitaceae, Cucurbitales)	109.1^1^	493
This study	*Epidendrum* (Orchidaceae, Asparagales)	*Phoenix dactylifera* (Arecaceae, Arecales)	114.6^1^	335
Mitchell et al. (2017)	*Protea* (Proteaceae, Proteales)	*Nelumbo nucifera* (Nelumbonaceae, Proteales)	117.4^1^	498
Cardillo et al. (2017)	*Hakea* (Proteaceae, Proteales)	*Nelumbo nucifera* (Nelumbonaceae, Proteales)	117.4^1^	450
Wanke et al. (2017)	*Aristolochia* subsec. *Pentandrae* (Aristolochiaceae, Piperales)	*Amborella trichopoda* (Amborellaceae, Amborellales)	139.4^1^	233

^1^Family divergence times taken from Magallón et al. (2015).

^2^Oldest crown-family divergence time reported in http://www.mobot.org/MOBOT/research/APweb.

* including paralogs.

Efficiency in terms of number of captured loci was relatively homogeneous across targeted species (335–314), except for *E. magnoliae* from which only 285 loci were recovered. Failure of capturing some loci for this species could be explained by the low percentage (the lowest among all the species) of reads on target, also reflected in its high percentage of missing data (see below; **Figure 1D**).

Regarding sequence quality, all species from subtribe Laeliinae, except *E. magnoliae* (9.5%), showed a relatively low percentage of missing data (6.8-1%), whereas the outgroup species *P. cardiothallis* from subtribe Pleurothallidinae showed a substantially higher proportion of missing data (12.5%). There is no evidence that genome sizes in Pleurothallidinae are larger than in Laeliinae (Leitch et al., 2019) and polyploidy has not been reported in the former subtribe (Felix and Guerra, 2010). Therefore, larger genome size or higher ploidy level seem unlikely to explain the lower enrichment efficiency for *P. cardiothallis*. Although genomic DNA of *P. cardiothallis* met similar quality standards as other species analyzed and was subjected to the same wet lab and bioinformatic processing, there may be lineage-specific variation in capture efficiency.

### Plastid Loci Mining Success

All four rRNAs and the 68 selected plastid protein encoding genes were successfully mined from off-target reads. Regarding the *ndh* gene family, our results agree with previous studies (e.g., Kim et al., 2015; Lin et al., 2015; Kim et al., 2017; Niu et al., 2017; Zhitao et al., 2017; Dong et al., 2018) which have shown that this set of genes are commonly pseudogenized, lost, or translocated in orchids, since most *ndh* genes for most of the target species were recovered most likely as pseudogenes and very rarely as functional genes. The *ndhA*, *ndhG*, *ndhH*, and *ndhI* genes were missing in one or several species; however, this cannot be assumed with confidence to be the consequence of gene loss, because there is a possibility that they were simply not recovered among the off-target reads. A comparative analysis of full *Epidendrum* plastomes could help to elucidate this in the future.

Our mining results are promising considering that the entire libraries were enriched with the target AHE nuclear markers and that a non-*Epidendrum* reference species (*M. coccinea* of subtribe Pleurothallidinae) was employed for extracting the plastid orthologs. We aimed at extracting plastid exons only, which are generally better conserved than non-coding regions such as introns or intergenic spacers (Shaw et al., 2005). Although not performed here, orchid studies aiming to extract the more variable non-coding plastid regions might be more challenging due to the expected higher sequence divergence between target and reference species. A way to overcome this would be using closer relatives as reference species. However, for *Epidendrum* this will only be possible when complete plastomes become available. To date, plastomes of 163 orchid species representing 46 genera have been sequenced (NCBI database; accessed June, 2019), therefore new orchid studies that apply our plastome mining approach will have a wealth of potential publicly available reference species. Another approach might be to perform further scaffolding to extract flanking non-coding regions, a strategy that we will follow in an upcoming publication.

It is remarkable that extremely long genes, such as *ycf2* (6,990 aligned bp), could be assembled and used for phylogenetic inference. Furthermore, most individual loci had a full taxon representation and the number of loci recovered per species was rather high (>70), except for *E. parkinsonianum* from which 68 loci were recovered. In general, percentage of missing data increased with the percentage of reads on target, or in other words, missing data increased if fewer non-target reads were available for assembling plastome regions.

### Phylogenetic Utility of Nuclear and Plastid Loci

Relationships here obtained among the included subtribe Laeliinae genera are mostly in agreement with those recovered by Hágsater and Soto-Arenas (2005) and van den Berg et al. (2009), but with stronger statistical support. Previous studies, all of them based on a few Sanger-sequenced loci, often failed to provide strong statistical support for intergeneric relationships, as well as for many internal relationships of *Epidendrum*. Our study overcame this limitation, albeit on a limited taxon sampling, by analyzing the largest set of nuclear and plastid loci generated to date for subtribe Laeliinae, with few nodes within *Epidendrum* receiving weak statistical support (BS < 85, **Figure 3**). *Caularthron* is sister to *Epidendrum* with strong support, in agreement with the results of van den Berg et al. (2009). Likewise, the two major clades recovered within *Epidendrum* in our analyses (marked as A and B in **Figure 3**) more or less correspond to the two main clades found by Hágsater and Soto-Arenas (2005), except for the placement in our trees of *E. nocturnum* in clade A instead of clade B. It is also worth noting that our data recover a strongly-supported clade of mostly Mexican species (*E. parkinsonianum*-*E. lacertinum* to *E. matudae*), which did not group but formed a grade at the base of clade B in the analysis of Hágsater and Soto-Arenas (2005). The five species of the *E. anisatum* group included here form a strongly-supported clade, in agreement with the various morphological and eco-geographical features they share (Quiroga-González, 2017), although the internal relationships in this clade include one incongruence between our plastid and nuclear trees, as discussed below (**Figure 4**).

Most of the resulting phylogenetic relationships were consistent across different analyses (summary and site-based) or data sources (plastid *vs*. nuclear). However, some strongly supported incongruences were detected when comparing the nuclear and plastid phylogenies. The higher number of strongly supported incongruences between the nuclear ASTRAL and the plastid tree (six incongruences), compared to those found between the nuclear SVDQuartets and the plastid tree (three incongruences), could be attributed to potential noise introduced by gene tree estimation error associated to summary methods (Roch and Warnow, 2015). Measures known to increase accuracy of ASTRAL analyses were herein applied, such as using input trees where nodes with BS < 33 are collapsed. However, nuclear loci varied widely in their aligned length, and differences in their number of potentially informative sites and their proportion of missing data could lead to estimation error in some short or highly incomplete alignments.

Because site-based methods are known to avoid difficulties associated to gene tree estimation, incongruences between the obtained nuclear and plastid hypotheses will be further discussed based on the SVDQuartets and RAxML trees. In these trees, incongruences involved recent divergences within *Epidendrum* corresponding to the alternative positions of *E. lacertinum*, *E. anisatum,* and *E. cusii*. When focused, for instance, on the alternative relationship of *E. matudae* as sister to *E. anisatum* in the plastid tree or as sister to *E. cusii* in the nuclear trees, we found that 46 (out of 335) nuclear gene trees recovered the alternative sister relationship between *E. matudae* and *E. anisatum* of the plastid tree. Although only three of these 46 nuclear gene trees recovered this relationship with high support (BS > 85), this indicates that the phylogenetic signal of the plastid hypothesis is shared with a small proportion of nuclear gene trees.

At these shallow evolutionary levels, processes such as incomplete lineage sorting may play a role if lineages are associated to deeper rapid radiations (Degnan and Rosenberg, 2009). A rapid radiation has not been formally tested in *Epidendrum*, but short internodes are characteristic of its phylogenetic tree (e.g., Hágsater and Soto-Arenas, 2005). Alternatively, branch length heterogeneity (in coalescent units) within *Epidendrum* observed in the nuclear ASTRAL tree may suggest potential changes of the effective population size across the evolutionary history of this genus, with short branches indicating increased gene tree discordance (Degnan and Rosenberg, 2009). A further potential source of conflict between biparentally and uniparentally inherited DNA data is hybridization and introgression. As revisited by Pinheiro and Cozzolino (2013), hybridization likely is a key process shaping the diversification of some groups of *Epidendrum*. Similar patterns of strongly-supported incongruence between nuclear and plastid partitions at shallow phylogenetic levels have been observed within most of the genera of Laeliinae that have been investigated using Sanger sequencing, e.g., *Cattleya* (van den Berg et al., 2009; van den Berg, 2014), *Encyclia* (Leopardi-Verde et al., 2017), and *Laelia* (Peraza-Flores et al., 2016). This seems to be caused by the combination of few genetic incompatibility barriers and very low variation in chromosome numbers across the subtribe, including *Epidendrum* (Felix and Guerra, 2010; De Assis et al., 2013), and confirmed by the large number of natural hybrids (including intergenerics) reported in the subtribe (Adams and Anderson, 1958; van den Berg, 2014).

Phylogenetic informativeness profiles allowed us to confirm a general trend that has been widely documented in vascular plant evolutionary studies, i.e., that nuclear data generally provides higher informativeness than plastid data, and that nuclear data better informs more recent evolutionary events than plastid data (e.g., Clegg et al., 1994; Sang, 2002; Duarte et al., 2010; Zimmer and Wen, 2012; Rothfels et al., 2013; Salas-Leiva et al., 2013; Soltis et al., 2013; Lu et al., 2014). Nuclear target *vs*. non-target regions contributed with similar levels of phylogenetic utility; however, nuclear non-target regions informed a wider range of more recent divergences compared to nuclear target regions.

### Phylogenetic Utility of the *ycf1* Gene at Low Taxonomic Levels

One remarkable exception to the general trend observed between plastid and nuclear markers is the *ycf1* gene, which by far surpassed the net informativeness of all other loci, either plastid or nuclear. Although until recently the possible function of *ycf1* was unknown (its gene abbreviation stands for hypothetical chloroplast open reading frame 1), mounting evidence indicates that it is part of an inner membrane envelope translocon, or TIC, i.e., a complex of proteins associated with the translocation of polypeptides across the inner membrane of the chloroplast (de Vries et al., 2015; Nakai, 2015). The *ycf1* gene alignment was the second longest of all the analyzed loci, only surpassed by the *ycf2* gene (see also Wicke et al., 2011), however, phylogenetic informativeness of *ycf2* is substantially lower than that of *ycf1*. To account for the differences in alignment length we calculated the per site phylogenetic informativeness (data not shown), finding that informativeness provided by the *ycf1* gene surpasses that of all plastid and several (40) nuclear partitions.

The unusually high phylogenetic utility of the plastid *ycf1* gene for shallow taxonomic levels in orchids was first highlighted by Neubig et al. (2009), who found two portions close to the 5’ and 3’ ends of this gene to be more variable than other genes commonly used for phylogenetic inference, such as *matK* and *rbcL*. Subsequently, this marker was used to inform phylogenetic relationships at low taxonomic levels in the orchid subtribes Oncidiinae (Chase et al., 2009; Neubig et al., 2012) and Maxillariinae (Arévalo et al., 2015), and partial (Gernandt et al., 2009; Drew and Sytsma, 2011; Drew and Sytsma, 2012; Drew and Sytsma, 2013; Shi et al., 2013) or entire (Parks et al., 2009) exon sequences of this gene have been used successfully to estimate phylogenetic relationships at intermediate to low taxonomic levels in other plant lineages. More recently, Dong et al. (2015) identified two segments of this gene as promising DNA barcodes for plants. Roma et al. (2018), in their study of the orchid genus *Ophrys,* attributed the unusually high sequence divergence of *ycf1* relative to other genes to its location in the junction of the inverted repeat and the small single copy regions, which additionally cause a high sequence length variation and potential pseudogenization (see also Jheng et al., 2012). Although our mining strategy does not provide information about gene location, it allowed us to compare the informativeness of complete sequences of most known plastid genes and confirm that the *ycf1* gene not only provides greater phylogenetic resolution than the commonly used *matK* and *rbcL* genes, as pointed out by Neubig et al. (2009) for orchids in general, but it is also more informative than any other plastid gene and several nuclear loci in *Epidendrum*. None of the species analyzed here showed signs of pseudogenization or loss of the *ycf1* gene. After its proposed origin before the diversification of green plants (Wicke et al., 2011), the *ycf1* gene is known to be absent in the Poaceae (Dong et al., 2015) and a few holoparasitic (*Orobanche purpurea*, Orobanchaceae) and photosynthetic eudicots (*Vaccinium macrocarpon*, Ericaceae, and *Erodium* spp., Geraniaceae; see de Vries et al., 2015), confirming it as an excellent candidate for phylogenetic inference at low taxonomic levels not only in *Epidendrum*, but in many other angiosperm lineages.

Integrating information from the chloroplast and nuclear genomes increased the range of evolutionary depths that could be estimated and contrasted in our study. It is worth noticing that, except for a few outliers, none of the analyzed partitions (target or non-target nuclear regions or plastid loci) had its maximum informativeness within the diversification time of subtribe Laeliinae. This is to be expected, because the inclusion of non-coding regions in our analyses was moderate. As explained in the *Materials and Methods* section, we did not aim to mine non-coding plastid regions, hence the only sources of non-coding data in our analyses were the adjacent non-target regions of the nuclear loci. A future study will focus on resolving shallower divergences among an expanded taxon sample of *Epidendrum*, by mining plastid non-coding regions to increase the resolution power at fine evolutionary levels using a newly sequenced *Epidendrum* plastome as reference.

## Conclusion

Our study demonstrated for the first time the technical implementation of the Angiosperm v.1 probe kit (Buddenhagen et al., 2016) to the orchid genus *Epidendrum*, supporting the universal applicability of this kit across angiosperms. Moreover, we confirmed the feasibility of mining plastome loci from off-target reads when using this kit, generating complementary sequence data of uniparental inheritance at no extra sequencing cost. Our analyses are in general congruent across methods and data sources. The few strongly supported incongruences detected suggest the possibility of incomplete lineage sorting or potential hybridization and introgression events among closely related species. Our ample survey of the phylogenetic utility of coding nuclear and plastid loci in *Epidendrum* allowed us to identify the *ycf1* gene as a strongly useful locus for resolving relationships at low taxonomic levels, surpassing the net informativeness of every other plastid and nuclear loci analyzed. Hyb-seq approaches (Weitemier et al., 2014) thus appear as a promising option for generating informative data sets derived from different genome compartments. Although our taxonomic sample is too small to attempt to draw conclusions about organismal and evolutionary aspects of the genus as a whole, our results provide a foundation for a much more inclusive sampling strategy aimed at covering the structural diversity of the genus throughout the Neotropics in a forthcoming phase of our research program.

## Data Availability Statement

Raw data associated to this article can be found under the NCBI Sequence Read Archive BioProject PRJNA589279.

## Author Contributions

GS, EH, CM, and SW conceived and designed the study. GS, EH, and CB designed the taxon sampling and collected or provided the samples. CM, EL, and AL performed the laboratory work. AL and MJ performed the bioinformatic process of nuclear and plastid data, respectively. CM performed the phylogenetic and informativeness analyses. CM drafted the manuscript and MJ, EH, SM, SW, CB, EL, AL, and GS proof read and approved the final manuscript.

## Funding

Funding for this research was provided by UNAM–DGAPA–PAPIIT project IG200316 and Fronteras de la Ciencia CONACYT project 2016-01-1867 (both to SM), and Instituto Chinoin, A.C. ERASMUS+ funding was granted to Technische Universität Dresden (TU Dresden) to support training mobility between Instituto de Biología, UNAM and TU Dresden.

## Conflict of Interest

The authors declare that the research was conducted in the absence of any commercial or financial relationships that could be construed as a potential conflict of interest.

## Supplementary Material

Supplementary Material S1 A .zip file containing the code, scripts and a description file (README.txt) used to generate the nuclear alignments from raw data.

Supplementary Material S2 Inferred nuclear gene trees.

Supplementary Material S3 A concatenated matrix of all nuclear loci partitioned by locus.

Supplementary Material S4 A concatenated matrix of all plastid loci partitioned by locus.

Supplementary Material S5 Table with number of raw reads and percentage of reads on target for each sampled species.

Supplementary Material S6 Resulting nuclear ASTRAL tree.

Supplementary Material S7 Resulting plastid RAXML tree.

Supplementary Material S8 Resulting nuclear SVDQuartets tree.

Supplementary Material S9 A figure comparing this and previous plant Anchored Hybrid Enrichment studies.
