# Supplementary figures and images for: Target Nuclear and Off-Target Plastid Hybrid Enrichment Data Inform a Range of Evolutionary Depths in the Orchid Genus Epidendrum
Source: Front Plant Sci. 2020 Jan 29;10:1761. doi: 10.3389/fpls.2019.01761 (PMC7000662; doi:10.3389/fpls.2019.01761)

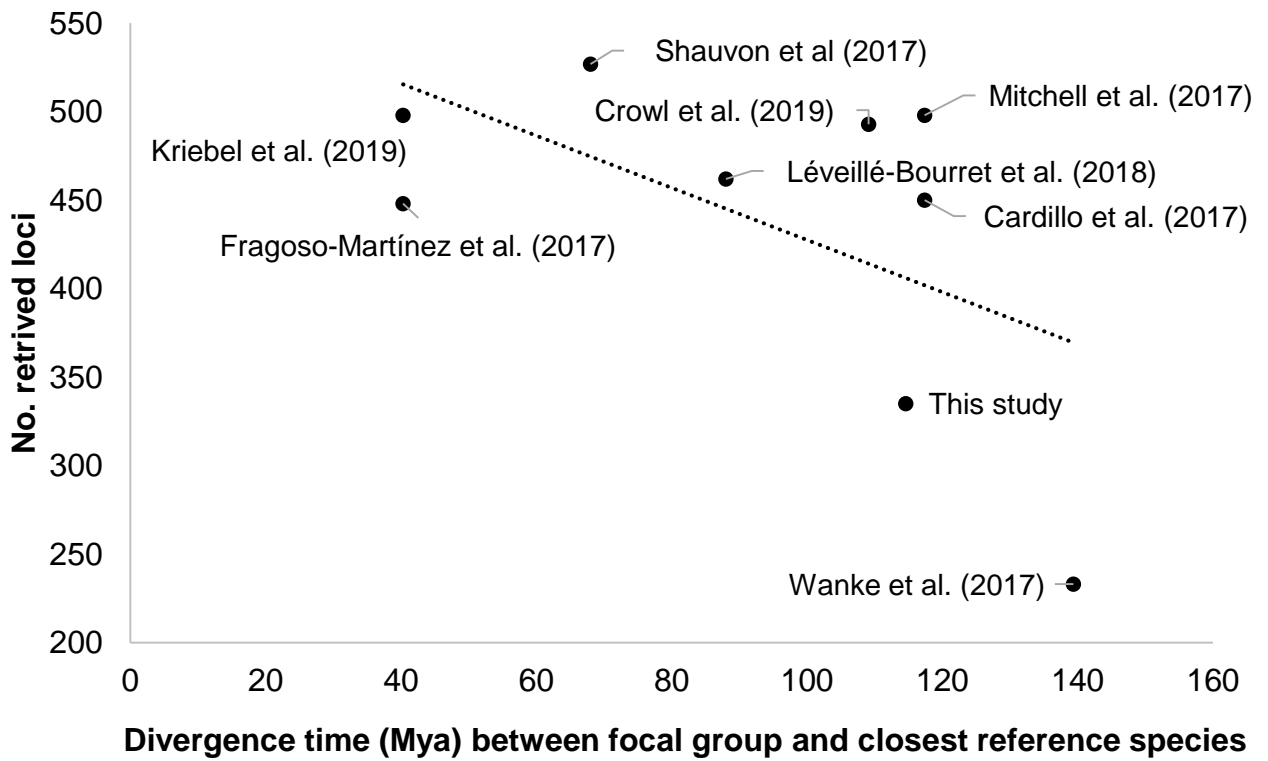

Supplement: Supplementary Material S1 — A .zip file containing the code, scripts and a description file (README.txt) used to generate the nuclear alignments from raw data. [file DataSheet_1.zip › Supplementary_Material_S9.pdf]
